# Tobacco and oral squamous cell carcinoma: A review of carcinogenic pathways

**DOI:** 10.18332/tid/105844

**Published:** 2019-04-12

**Authors:** Xiaoge Jiang, Jiaxin Wu, Jiexue Wang, Ruijie Huang

**Affiliations:** 1 Department of Pediatric Dentistry, State Key Laboratory of Oral Diseases, National Clinical Research Center for Oral Diseases, West China Hospital of Stomatology, Sichuan University, Chengdu, China

**Keywords:** tobacco, smoking, oral squamous cell carcinoma, carcinogenic pathways

## Abstract

**INTRODUCTION:**

Tobacco is one of the most important risk factors for premature death globally. More than 60 toxic chemicals in tobacco can invade the body’s various systems. Oral squamous cell carcinoma (OSCC) is a pathological type of oral cancer, accounting for over 90% of oral cancers. A vast quantity of scientific, clinical and epidemiological data shows that tobacco is associated with the development of oral squamous cell carcinoma, and its carcinogenic pathways may be complicated.

**METHODS:**

We conducted a thorough electronic search by Cochrane, EMBASE and PubMed to identify relevant studies. Studies published up to the end of October 2018 were included. After assessing and selecting articles based on eligibility criteria, studies were classified and elaborated according to the pathogenesis.

**RESULTS:**

Tobacco as an important risk factor can cause epigenetic alteration of oral epithelial cells, inhibit multiple systemic immune functions of the host, and its toxic metabolites can cause oxidative stress on tissues and induce OSCC. In addition, some specific viruses such as EBV and HPV are thought to play a role in the development of OSCC.

**CONCLUSIONS:**

Oral cancer ranks eighth among the most common causes of cancer-related deaths worldwide, and tobacco is one the most important carcinogenic factors of OSCC. This review of the literature attempts to provide directions and ideas for future related research, and emphasizes the need for efforts to reduce tobacco consumption.

## INTRODUCTION

It is well known that tobacco is one of the most important risk factors for premature death globally^1^. It is reported that there are more than 1.3 billion smokers worldwide^2^. The World Health Organization (WHO) estimates that tobacco causes nearly 6.4 million deaths and hundreds of billions of dollars of economic damage worldwide each year^3^. If current trends continue, by 2030 tobacco will kill more than 8 million people worldwide each year, most of which will occur in developing countries with lower incomes^4^. Although many people are aware that tobacco harms their health, most still accept smoking as part of their daily life, unaware that more than 60 toxic chemicals including carcinogens and cancer-promoting substances^5,6^, in tobacco can invade the body’s various systems^7^. Each cigarette is made of many ingredients, and some tobacco companies may use certain flavor additives to make their tobacco products more attractive, which may also be harmful to health^8^. Not only can these original components cause harm, but the intermediate metabolites play an unavoidable role in the process during smoking.

Oral squamous cell carcinoma (OSCC) is a pathological type of oral cancer, accounting for over 90% of oral cancers^9^. Oral cancer ranks eighth among the most common causes of cancer-related deaths worldwide^10^. Oral and oropharyngeal cancers are reported to account for approximately 220000 new cases per year (5% of all cancers) worldwide^11^. According to the recent epidemiology of OSCC, the incidence in lower/middle income countries or developing countries tends to be higher than that of developed countries^12^. The data show that the risk factors that attribute to OSCC are age, sex, race, gender, tobacco, alcohol, betel nut, diet and nutrition^13^. Among them the most common is tobacco. Many epidemiological studies have demonstrated a clear dose-response relationship between tobacco use and the risk of oral cancer or potentially malignant oral disease. Early in 1994, a study^14^ analyzed 454 patients with oral carcinoma and found that 60% of those with oral carcinoma smoked and over 95% of neoplasms were squamous cell carcinoma, while another study^15^ in 1999 stressed the significance of tobacco in the progress of oral epithelial dysplasia (OED) in a large number of European patients.

More than 180000 cases of oral cancer occur every year in South-East Asia; approximately 90% of which are due to smoking and chewing habits. Depending on the different products, tobacco may contain more than 60 established or potential carcinogens that can increase the relative risk of cancer through different mechanisms, including oxidative stress on tissues, persistent reactive oxygen species, lipids, carbohydrates and DNA to disrupt cell cycle-regulated mutations or through effects on the immune system^16^.

It is widely accepted that tobacco is one the most important carcinogenic factors of OSCC, and its carcinogenic pathways may be multifaceted. The purpose of this review is to summarize the possible mechanisms of tobacco that promote the development of OSCC, on the basis of relevant research, so as to provide directions and ideas for future related research.

## METHODS

### Eligibility criteria

The eligibility criteria for studies were: 1) research articles that studied the pathogenesis of oral squamous cell carcinoma (SCC) caused by smoking, and 2) articles in English. Epidemiological investigations, reports, literature reviews, comments and letters to the editor were excluded.

### Search strategy and studies selection

We conducted a thorough electronic search by Cochrane, EMBASE and PubMed to identify relevant studies. Studies published up to the beginning of December 2018 were included. The search terms were: [Mouth Neoplasms OR Oral carcinoma OR Oral squamous cell carcinoma OR Oral Sprays OR OSCC] AND [Tobacco OR Smoking OR Tobacco Products OR Tobacco smoking OR Cigarette OR Cigarette smoking OR Cigar]. No data restrictions were applied in searching.

## RESULTS

**Figure 1:**
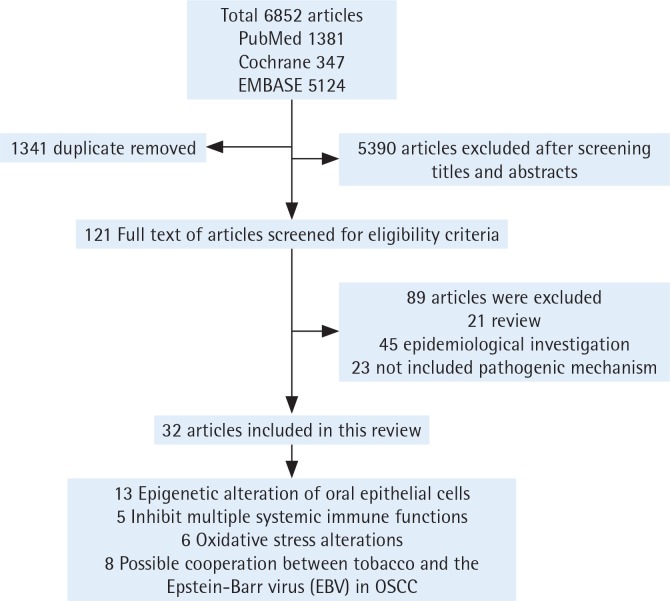
Flow chart of studies selection process

We first excluded duplicate articles. Then, two authors independently assessed the titles and abstracts of the studies on the basis of the theme of this review. Next, the full text of the remaining studies was evaluated and articles without conclusions of pathogenesis were excluded. Finally, studies were classified and elaborated according to the pathogenesis. When the two authors’ opinions were not uniform, consensus was reached through discussion along the process (Figure 1).

## DISCUSSION

### Possible carcinogenic pathways

**Figure 2:**
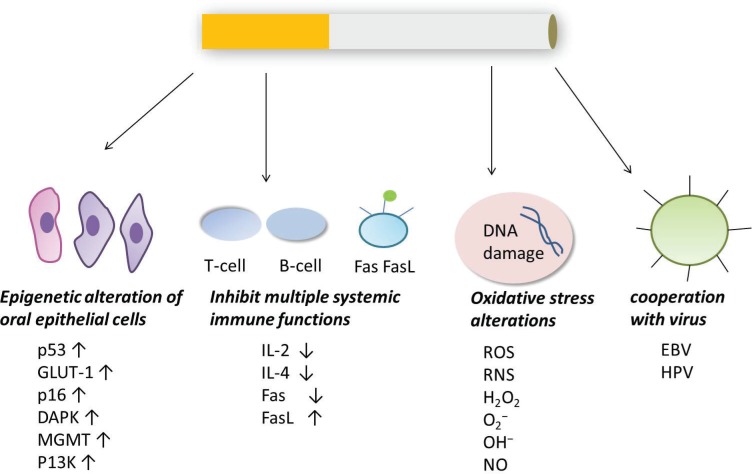
The possible carcinogenic pathways

The possible carcinogenic pathways are summarized in Figure 2.

#### Epigenetic alteration of oral epithelial cells

Many studies have shown that tobacco can cause the abnormal expression of p53, GLUT-1, p16, DAPK, MGMT, P13K and other genes in oral epithelium, which is associated with the occurrence of OSCC.

The p53 cancer suppressor gene is the most universally identified mutated gene in human malignancies. The protein encoded by it is a transcriptional factor that controls the start of the cell cycle^17^. The p53-mediated cellular signal transduction pathway plays an important role in regulating normal cell life activities. After mutation, the p53 gene loses its regulatory effects on cell growth, apoptosis and DNA repair, and transforms from a tumor suppressor gene into an oncogene^18^. In 1994^19^, a study in India investigated the expression of p53 protein in premalignant oral lesions and observed that p53 aberrations are an inchoate change in the development of oral carcinoma. They found that the proportion of p53 protein overexpression was high in premalignant and malignant oral lesions in patients who were heavily consuming tobacco. Later, further studies^20-23^ verified that tobacco was associated with the overexpression of the p53 gene in epithelial cells.

Glucose transporters (GLUTs) are a protein family that mediates glucose transport through the cell membrane. Glucose metabolism depends on the uptake of glucose by cells. However, glucose cannot freely enter cells through the lipid bilayer structure of the cell membrane. Glucose uptake by cells requires glucose transporters on the cell membrane. The expression of GLUT-1 is upregulated in malignant cells, which suggests increased proliferative activity, energy requirements, aggressive behaviour and poor radiation response^24^. GLUT-1 expression correlates significantly with histological grade and pathology Tumor Node Metastasis (pTNM) staging of OSCC^25-27^. A recent study showed that GLUT-1 significantly correlates with tobacco-related human oral carcinoma^28^. In that study, involving 50 samples, the tobacco addiction group showed a larger proportion of cells displaying GLUT-1 immunostaining (79.2%) compared with the non-tobacco group (52%), which was statistically significant.

In addition, p16 (MTS, multiple tumor suppressor 1), DAPK (death-associated protein kinase), MGMT (O6-methylguanine-DNA methyltransferase), PI3K (the phosphatidylinositol 3-kinase), c-myc and other genes were investigated in oral tobacco-related tumor tissues and cancer associated adjacent tissues^21,29-32^. The epigenetic alteration of these genes is a common event in oral malignancy, and is an inchoate change discovered in oral mucosa of these patients. It indicates that epigenetic alteration is of vital importance in tobacco associated oral carcinogenesis. To validate the findings, further studies are needed that comprise larger sample sizes.

#### Inhibition of multiple systemic immune functions

Immune dysfunction plays an important role in the escape of cancer cells from effector immunological functions, leading to the occurrence, establishment and development of the cancer. The incidence of malignancy in immunocompromised patients is 100 times higher than in normal ones^33^.

IL-4 is an anti-inflammatory cytokine and various in vitro studies have documented its anti-tumor activity on breast and colon cancer^34^. It directly modulates proliferation of various cancer cell types including gastric and renal cancers by increasing expression of p21WAFI and interferon regulating factor (IRF-1) and decreasing cyclin-dependent kinase (CDK)-2 activities besides facilitating the infiltration of inflammatory cells such as macrophages, eosinophils, and neutrophils^35^. A study^36^ in 2010 investigated the systemic immunity and the expression of IL-4 and IL-2 in T-cell subsets from peripheral blood of tobacco-related OSCC patients, on the basis of major lymphocyte subsets. They found that those with oral malignancy showed obviously decreased CD4+ and CD3+ T-cell subsets with a lower CD4/ CD8 percentage in comparison to the normal controls. The proportion of CD4+ IL-2+ was obviously lower while CD8+ IL-4+ and CD3+ IL-4+ T cells were significantly higher in them in comparison to the normal controls. Decreased expression of IL-2 in both CD8+ and CD4+ subsets was connected with the late stage of the neoplasm. The tobacco-related oral cancer is likely to be connected with multiple systemic immune impairs, especially defected CD4+ and CD3+ T cells and a differential regulation of IL-4 and IL-2 in CD8+ and CD4+ T-cell subsets in the peripheral blood. In addition, some scientists assessed the relationship between IL-4 promoter and IL-6 functional genetic polymorphisms in Asian Indians and tobacco-related oral cancer. In the study^37^, IL-4 genotype seemed to be susceptible in patients, while IL-6 genotype seemed to be protective. It is suggested that tobacco can decrease the transcription rate of IL-4 gene as this may have anti-tumor effects.

Fas receptor and Fas Ligand (FasL) system are associated with the suppression of apoptosis, insensitivity to chemotherapy, and with providing immune privilege to a majority of the tumours via the Fas mediated apoptosis of tumour-specific lymphocytes^38-40^. The decreased expression of Fas and/or increased expression of FasL avails tumour transformation and malignant progression^41^. A study^42^ in 2011 used DNA flow cytometry for cell cycle parameters and immunohistochemistry for Fas and FasL on 10 normal samples and 41 paraffin embedded tumours. The results showed that low Fas expression was observed only in 2 of 41 (5%) oral tumours while FasL immunoreactivity was observed in 26 of 41 (63.4%) tumours on the cell membrane. In contrast, all 10 normal oral tissues performed strong cytoplasmic and membrane Fas receptor immunoreactivity but without FasL staining. Up-regulation of FasL and downregulation of Fas receptor is likely to be an important character of tobacco-related OSCC.

From the above, the immunosuppressive effects may exist in tobacco-related OSCC. The IL-4 promoter and IL-6 functional genetic polymorphisms, Fas and FasL system etc. are supposed to be used as important prognostic variables in tobacco-related OSCC patients. Further, the IL-1β-511 C/T polymorphism^43^, interferon(IFN)^44^ may also play a role. Future studies that include larger sample sizes are needed to validate these findings.

#### Oxidative stress alterations

Tobacco, which is a foreign substance^45^, has been shown to stimulate^46^ the body to produce more free radicals that are endogenously produced in various cellular metabolic activities and which play a role in preventing microbial pathogen invasion at low concentrations. However, as their concentration rises, they may damage cellular components, ultimately leading to denaturation or mutation, which can be seen in parasitic infections, inflammatory diseases and cancers^47^. It has been proven that oral cancer is related to oxidative stress^48,49^. Free radicals include reactive oxygen species (ROS), reactive nitrogen species (RNS) and reactive oxygen metabolites such as hydrogen peroxide (H_2_O_2_), superoxide anions (O2-), hydroxyl radicals (OH-), nitric oxide (NO) and malondialdehyde. They can induce several DNA damages including strand breakage, DNA-protein cross-linkage and base modification. They can form lipid peroxides and react with cell membrane fatty acids^45^. For instance, ROS and RNS participates in the initiation and promotion of carcinogenesis through DNA damage^50,51^. NO-mediated base excision inhibits DNA repair, which may aggravate oxidative DNA damage in cells, which is possibly related to carcinogenesis^52,53^. They can also affect antioxidant systems. For example, GSH^54^ levels are a key factor in protecting organisms from toxicity and disease, as they provide reduced power for several reactions and play an important role in the detoxification of hydrogen peroxide and other free radicals. Higher oxidants and lower antioxidant activities in blood of cancer cases suggest their importance in progression of disease^55,56^. They can also decrease the normal effects of the superoxide dismutase (SOD).

A study^57^ in 2005 researched tobacco habits and alterations in enzymatic antioxidant system in oral cancer. They found that the risk of oral cancer development in smokers was significantly higher than non-smokers on the basis of erythrocytic glutathione reductase (GR), SOD, catalase (CAT) and plasma thiol. In addition, updated studies^58-62^ have proven the association between tobacco related OSCC and oxidative stress alterations. More research is needed to validate these findings.

#### Possible cooperation between tobacco and the Epstein– Barr virus (EBV) in OSCC

Epstein-Barr virus (EBV), also known as human herpes virus 4 (HHV-4)^63^, is a type of herpes virus^64^. EBV causes lifelong persistent infections in more than 90% of the world’s population. The virus has an incubation period in healthy individuals carrying the virus. Under ambient pressure, the virus can be reactivated periodically during the lifetime of the individual. EBV is often associated with a variety of malignancies such as Burkitt’s lymphoma, Hodgkin’s disease, stomach cancer, and nasopharyngeal carcinoma (NPC)^65^.

Tobacco is an important risk factor, which through its toxic metabolites, can cause DNA damage that induces OSCC. In addition, some specific viruses are thought to play a role in the development of OSCC^66^. For example, a study showed that there is a possible interaction between tobacco and HPV16 in inducing OSCC^67^. Some published controlled studies provide indirect evidence of a significant epidemiological association between EBV and OSCC^68^. Furthermore, several EBV proteins have been found to be expressed in OSCC tissues and are associated with tumor phenotypes^69^, which indicates that there is a strong correlation between EBV and OSCC. As mentioned above, the relevant pathogenesis of NPC is similar. Some authors have noticed the association between tobacco and EBV through epidemiological investigations, and they believe that tobacco may play a role in the carcinogenesis of NPC by inducing EBV reactivation. In a study^70^ in the Guangdong province of China, it was shown that cigarette smoke extract (CSE) promotes EBV replication in Akata and B95-8 cells and enhances the expression of the EBV transcriptional factors, Zta and Rta, which may be the latent-to-lytic switch of EBV. It confirmed that tobacco may act as an inducer of recurrent EBV reactivation^71^, which has been shown to promote genome instability and enhance NPC progression. However, few studies have mentioned the interaction of EBV and tobacco in the development of OSCC^72^, or in other words, provided sufficient evidence to explain the relationship between them. The results of one study showed that in OSCC patients, the difference in EBV prevalence between the smoking control group and the non-smoking control group was not significant. We believe that EBV is present in oral diseases such as OSCC and OLP. Smoking, drinking or age does not appear to be a risk factor for EBV infection. However, another study in Yemen^73^ showed a strong correlation between the rate of exposure to shama (tobacco) and the positive rate of EBV in OSCC patients. This has never been reported before. In conclusion, it can be assumed that tobacco induces the occurrence of OSCC by inducing EBV reactivation, similar to NCP. This carcinogenic pathway may be another potential mechanism. Further epidemiological and experimental studies are necessary to confirm the interaction between tobacco use and EBV positivity in oral cancer and to reveal potential mechanisms.

## CONCLUSIONS

It has been proven that the use of tobacco is associated with the development of OSCC. Based on existing research, tobacco can cause epigenetic alteration of oral epithelial cells, inhibit multiple systemic immune functions of the host, and through its toxic metabolites cause oxidative stress on tissues to induce OSCC. In addition, some specific viruses such as EBV and HPV are thought to play a role in the development of OSCC. To validate these findings, further studies are needed comprising larger sample sizes. Meanwhile, with the development of research on this topic, more possible mechanisms remain to be studied. As the treatment of OSCC is difficult and the prognosis is poor, further research on this topic will be helpful for early diagnosis or prevention of tobacco-related oral carcinoma through efforts for cessation of tobacco consumption.
